# *Roseomonas gilardii* in patient with leukemia and acute appendicitis: case report and review

**DOI:** 10.11604/pamj.2020.36.283.24834

**Published:** 2020-08-14

**Authors:** Francesk Mulita, Nikoleta Oikonomou, Athanasios Provatidis, Agelos Alexopoulos, Ioannis Maroulis

**Affiliations:** 1 Department of General Surgery, General University Hospital of Patras, Patras, Greece,; 2 Department of Pediatrics, Neonatal Intensive Care Unit, General University Hospital of Patras, Patras, Greece,; 3 Department of Hematology, General University Hospital of Patras, Patras, Greece

**Keywords:** Appendicitis, acute lymphoblastic leukemia, *Roseomonas gilardii*

## Abstract

Appendicitis is one of the most common abdominal conditions requiring emergency surgery. However, acute appendicitis in patients with leukemia is a rare condition. We report herein the case of an 18-year-old female with acute lymphoblastic leukemia (ALL), who was hospitalized in hematology department because of abdominal pain and fever. Ultrasound (US) of the abdomen revealed appendicitis and the patients underwent open appendectomy. The patient recovered without complications and was discharged in a good condition. The day of the operation blood and peritoneal fluid cultures were taken and Roseomonas gilardii was detected and healed empirically. The correct diagnosis of appendicitis in patients with leukemia and their management is challenging for physicians. Very rare microorganisms can be detected in these patients.

## Introduction

Appendicitis is one of the most common abdominal conditions in both adults and children requiring emergency surgery. However, acute appendicitis in patients with leukemia is uncommon and often recognized late. The correct diagnosis of appendicitis in patients with leukemia is challenging, because the clinical presentation is nonspecific and can be easily confused with the symptoms of leukemia and/or chemotherapy for this disease. Until now, only a few cases of appendicitis in patients with leukemia have been reported [1]. Appendectomy is used with caution in patients with leukemia, who are at an increased risk of complications and death [2]. We herein report a case of an 18-year-old female with acute lymphoblastic leukemia (ALL) and acute appendicitis.

## Patient and observation

**Figure 1:**
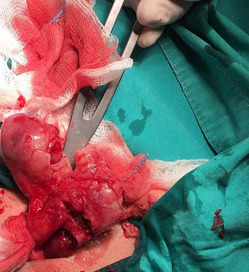
open appendectomy through a right lower abdominal incision

An 18-year-old female with acute lymphoblastic leukemia (ALL) was hospitalized in hematology department because of abdominal pain for 24 hours which was not associated with heartburn or vomiting. On examination, the patient´s temperature was 38.4, heart rate was 85 beats per minute, blood pressure was 115/71 and respiratory rate was 14 breaths per minute. Her abdomen was soft, without distension and only mild tenderness in the lower right quadrant was found. The initial haemoglobin was 9.46g/dL, white blood cells were 1.55K/μl, platelets were 58.800K/μl and C-reactive protein level was 1.35U/l. Liver and renal function test and serum amylase were normal. According to the real time ultrasound (US) of the abdomen which was performed immediately, the diameter of the appendix was found 9mm as well as hyperechoic mucosa and free fluid surrounding appendix was recognized. The patient underwent open appendectomy through a right lower abdominal incision (Figure 1). She had no intra-operative or postoperative complications. On day 3 of surgery, patient was discharged in good condition and referred to hematology department for further management. Her final histopathology report was suggestive of acute appendicitis. In addition, *Roseomonas gilardii* was detected from blood and peritoneal fluid cultures that were taken the day of the operation.

## Discussion

Acute lymphoblastic leukemia is a malignant disease of the bone marrow, in which a lymphoid precursor proliferates and replaces the normal hemopoietic cells of the marrow. Lymphoid precursor cells are arrested in an early stage of development. This arrest is caused by an abnormal expression of genes, often as a result of chromosomal translocations or abnormalities of chromosome number. Acute lymphoblastic leukemia is the most common type of cancer in children in United States. Median age at diagnosis is 16 years. There are two major types of acute lymphoblastic leukemia: Precursor B cell lymphoblastic leukemia and Precursor T cell lymphoblastic leukemia [3-4]. Clinical manifestations of acute lymphoblastic leukemia include constitutional symptoms such as fatigue, fever, and weight loss. Other clinical manifestations are the signs and symptoms of anemia (such as pallor, fatigue, dizziness, palpitations, cardiac flow murmur), the signs and symptoms of thrombocytopenia (such as bleeding, petechiae, ecchymoses), palpable lymphadenopathy, splenomegaly, rashes from skin infiltration with leukemic cells and frequent infections.

The diagnosis of acute lymphoblastic leukemia is based on laboratory tests from the peripheral blood, imaging studies and bone marrow studies. Laboratory tests from the peripheral blood include complete blood count, coagulation studies, peripheral blood smear, chemistry profile including lactate dehydrogenase, uric acid, liver function studies, and BUN/creatinine. Bone marrow studies include histology, immunohistochemistry/flow cytometry, cytogenetics, fluorescence in situ hybridization and PCR. Generally, the treatment of acute lymphoblastic leukemia includes the following stages: Induction chemotherapy, consolidation chemotherapy, maintenance chemotherapy, intrathecal chemotherapy for central nervous system (CNS) prophylaxis. Of course, treatment is individualized, based on clinical parameters such as the patient age (adolescent and young adult versus older) and on laboratory parameters such as Philadelphia chromosome status (positive versus negative). It is common clinical practice the inclusion of rituximab for CD20-positive patients (especially those < 60 years) and the inclusion of tyrosine kinase inhibitors for Philadelphia chromosome-positive (Ph+) disease. In current practice, most adult patients (except the most elderly and those with significant comorbidities) are offered allogeneic transplantation in first remission [5].

Traditionally, patients with acute lymphoblastic leukemia are divided into three prognostic groups: good risk, intermediate risk, and poor risk, based on parameters such as: cytogenetics, age, white blood cell (WBC) count and complete remission within 4 weeks. Good-risk criteria are considered the following: No adverse cytogenetics, age younger than 30 years, white blood cell (WBC) count of less than 30,000/μL and complete remission within 4 weeks, whereas poor-risk criteria are considered the following: adverse cytogenetics such as translocations t (9;22), t(4;11), age older than 60 years , precursor B-cell WBCs with WBC count greater than 100,000/μ, and failure to achieve complete remission within 4 weeks. The addition of tyrosine kinase inhibitors to chemotherapy has resulted in improved prognosis of patients with Philadelphia chromosome. Unfortunately, only 20-40% of adults with acute lymphoblastic leukemia are cured with current treatment regimens [6]. *Roseomonas gilardii* is a species of Gram negative, aerobic, pink-pigmented coccobacillus bacterium. It belongs to the type species of the genus Roseomonas. A feature of this organism is its slow-growth properties on culture; it often takes 4 to 5 days before any growth is seen. Human infections caused by *Roseomonas* are very rare.

There are only a few reports in the bibliography regarding infections by *Roseomonas gilardii*. It is considered to be an opportunistic pathogen which can lead to infections especially in immunosuppressed individuals and in patients with underlying medical illness such as acquired immune deficiency syndrome, chronic renal disease or diabetes mellitus. The most common clinical presentation is bacteremia and may be related to the presence of intravascular catheters, but it is not always the case [7-8]. Other clinical presentations are peritoneal dialysis-associated peritonitis and osteomyelitis, septic arthritis. Cranioplasty infection and nosocomial ventriculitis due to *Roseomonas gilardii* complicating subarachnoid haemorrhage have also been described [9]. Infections by *Roseomonas gilardii* are quite difficult to threat. This bacterium considered to be multi-drug resistant. Penicillins and cephalosporins (including ceftriaxone, ceftazidime) appear ineffective against any of the *Roseomonas* species. Carbapenems, aminoglycosides, tetracyclines and fluoroquinolones are the most active antibiotics against Roseomonas species. All patients are usually healed with empirical antibiotic therapy [9-10]. In our case the isolate was susceptible to amikacin, amoxicillin/clavulanic acid, cefepime, ceftriaxone, ciprofloxasin, gentamicin, imipenem, netilmicin, sulfomethoxazole-trimethoprime, tobramycin and resistant to ampicilin, cefuroxime, ceftazidime, piperacillin-tazobactam.

## Conclusion

In our rare case we reported a patient with leukemia who underwent open appendectomy and *Roseomonas gilardii* was detected from blood and peritoneal fluid cultures that were taken the day of the operation. The bacterium was healed with empirical antibiotics and patient was discharged in a good condition. The correct diagnosis of appendicitis in patients with leukemia and their management is challenging for medical professionals. Very uncommon microorganisms can be detected in these patients and early diagnosis is very important.

## References

[1] Wang C, Huang H-Z, Yu Y-J, He Y, Han S-L (2019). Acute Appendicitis in Patients with Leukemia: A Dilemma in Diagnosis and Surgical Treatment. Clin Surg.

[2] Kim EY, Lee JW, Chung NG, Cho B, Kim HK, Chung JH (2012). Acute appendicitis in children with acute leukemia: experiences of a single institution in Korea. Yonsei Med J.

[3] Arber DA, Orazi A, Hasserjian R, Thiele J, Borowitz MJ, Le Beau MM (2016). The 2016 revision to the World Health Organization classification of myeloid neoplasms and acute leukemia. Blood.

[4] Campo E, Swerdlow SH, Harris NL, Pileri S, Stein H, Jaffe ES (2011). The 2008 WHO classification of lymphoid neoplasms and beyond: evolving concepts and practical applications. Blood.

[5] Brown P, Inaba H, Annesley C, Beck J, Colace S, Dallas M (2020). Pediatric Acute Lymphoblastic Leukemia, Version 2.2020. NCCN Clinical Practice Guidelines in Oncology. Journal of the National Comprehensive CancerNetwork: JNCCN.

[6] Igwe IJ, Yang D, Merchant A, Merin N, Yaghmour G, Kelly K (2017). The presence of Philadelphia chromosome does not confer poor prognosis in adult pre-B acute lymphoblastic leukaemia in the tyrosine kinase inhibitor era-a surveillance, epidemiology, and end results database analysis. Br J Haematol.

[7] Srifuengfung S, Tharavichitkul P, Pumprueg S, Tribuddharat C (2007). Roseomonas gilardii subsp rosea, a pink bacterium associated with bacteremia: the first case in Thailand. Southeast Asian J Trop Med Public Health.

[8] Shokar NK, Shokar GS, Islam J, Cass AR (2002). Roseomonas gilardii infection: case report and review. J Clin Microbiol.

[9] Ece G, Ruksen M, Akay A (2013). Case report: cranioplasty infection due to Roseomonasgilardii at a university hospital in Turkey. Pan Afr Med J.

[10] Dé I, Rolston KV, Han XY (2004). Clinical significance of Roseomonas species isolated from catheter and blood samples: analysis of 36 cases in patients with cancer. Clin Infect Dis.

